# Characterizing multisegment foot kinematics during gait in diabetic foot patients

**DOI:** 10.1186/1743-0003-6-37

**Published:** 2009-10-23

**Authors:** Zimi Sawacha, Giuseppe Cristoferi, Gabriella Guarneri, Stefano Corazza, Giulia Donà, Paolo Denti, Andrea Facchinetti, Angelo Avogaro, Claudio Cobelli

**Affiliations:** 1 Department of Information Engineering, University of Padova, Italy; 2 Department of Clinical Medicine & Metabolic Disease, University Polyclinic, Padova, Italy

## Abstract

**Background:**

The prevalence of diabetes mellitus has reached epidemic proportions, this condition may result in multiple and chronic invalidating long term complications. Among these, the diabetic foot, is determined by the simultaneous presence of both peripheral neuropathy and vasculopathy that alter the biomechanics of the foot with the formation of callosity and ulcerations. To diagnose and treat the diabetic foot is crucial to understand the foot complex kinematics. Most of gait analysis protocols represent the entire foot as a rigid body connected to the shank. Nevertheless the existing multisegment models cannot completely decipher the impairments associated with the diabetic foot.

**Methods:**

A four segment foot and ankle model for assessing the kinematics of the diabetic foot was developed. Ten normal subjects and 10 diabetics gait patterns were collected and major sources of variability were tested. Repeatability analysis was performed both on a normal and on a diabetic subject. Direct skin marker placement was chosen in correspondence of 13 anatomical landmarks and an optoelectronic system was used to collect the data.

**Results:**

Joint rotation normative bands (mean plus/minus one standard deviation) were generated using the data of the control group. Three representative strides per subject were selected. The repeatability analysis on normal and pathological subjects results have been compared with literature and found comparable. Normal and pathological gait have been compared and showed major statistically significant differences in the forefoot and midfoot dorsi-plantarflexion.

**Conclusion:**

Even though various biomechanical models have been developed so far to study the properties and behaviour of the foot, the present study focuses on developing a methodology for the functional assessment of the foot-ankle complex and for the definition of a functional model of the diabetic neuropathic foot. It is, of course, important to evaluate the major sources of variation (true variation in the subject's gait and artefacts from the measurement procedure). The repeatability of the protocol was therefore examined, and results showed the suitability of this method both on normal and pathological subjects. Comparison between normal and pathological kinematics analysis confirmed the validity of a similar approach in order to assess neuropathics biomechanics impairment.

## Background

The chronic hyperglycemia of diabetes, a highly widespread chronic disease, is associated with long-term damage, dysfunction, and failure of various organs. In particular, patients experience neuropathy and blood vessels degeneration. These two complications develop into the foot disease which alters the biomechanics of gait and eventually leads to the formation of callosity and ulcerations.

The social and economic burden of the diabetic foot can be reduced through early diagnosis and treatment. Diabetic neuropathy is present in 25% of the patients after 10 years of disease, and it is the most significant risk factor for the development of foot ulcers. It consists in the distal symmetrical polyneuropathy which affects the motor and sensitive systems, both involved in the pathogenesis of the diabetic foot [1]. The motor sensitivity deficit exposes the patient to the risk of ulcers [2]. In addition, motor neuropathy leads to the degeneration of intrinsic foot muscles (lumbrical and interosseous) that cause deformation of the metatarsal heads and, in turn, excessive plantar loads during gait that predispose to callus formation, hyperkeratosis and ulcers [3]. Callus formation is associated with biomechanical foot dysfunction especially abnormal subtalar joint pronation [4].

Given this scenario, it is of paramount importance to study diabetic foot biomechanics in order to assess the risk of ulceration. These patients exhibit a displacement of the fulcrum of the step from the tibio-tarsal to the coxo-femoral joint, and an increase of the base of support with ataxic posture together with posture modifications [5-8]. The additional alterations of the soft tissues, tendons and the ligaments lead to a further limited joint mobility occurring especially to 1^st ^metatarsophalangeal and subtalar joints [4]. In particular the plantar fascia behaves like one rigid lever during the step, reducing the adaptability to the ground [9-12].

The study of structure and function of the diabetic foot have received little attention in the literature, while most of the studies have concentrated on the kinetic analysis by means of force and plantar pressure plates [4-12]. On the other hand kinematic analysis would be clinically very important for diabetic neuropathic patients in order to appreciate the supination-pronation and inversion-eversion movement of forefoot vs midfoot and hindfoot. Unfortunately, currently available movement analysis protocols [13-16] are not suitable for this purpose. These procedures, which utilize rigid mounting plates by means of elastic bandages and lengthy anatomical calibration procedures [13-15,17], cannot be easily applied in patients with peripheral artery disease or neuropathies. Protocols which consider the foot as a single rigid segment or does not consider the motion of the midfoot relatives to the adjacent subsegments cannot fully describe the diabetic foot disease consequences [13-22]. Therefore direct skin marker placement on selected anatomical landmarks (ALs), was chosen, together with a 3D four segments foot kinematics protocol. A static acquisition was used to define the anatomical Bone Embedded Frames (anatomical BEFs). Diabetic patients frequently have rigidity of toes or presence of ulcers which make protocols requiring marker placement on hallux impossible [13,18,19,21,22]. Moreover, the most recent studies [19,21,22] do not report all three rotational degrees of freedom of the three relevant foot sub-segments. Foot biomechanics alteration in the neuropathic patients [4] affects also their posture [6,23], this entails that a foot motion analysis protocol must be incorporated in a full body gait analysis protocol [15-17,20,24-26]. Finally, no study has reported on the clinical impact of foot kinematic analysis in diabetic patients [13,14,18,19,21,22].

The objective of this study was to devise a reproducible and clinically meaningful protocol [25] specific for the treatment of diabetic patients, which starting from the kinematics could help in preventing diabetic foot from ulcer or callus formation.

## Methods

Experiments were carried out using a six camera stereophotogrammetric system (BTS, Italy) with a sampling rate of 60 frames per second synchronized with two Bertec force plates (FP4060-10). Force plates were used to determine the gait cycle parameters (time and space). Ten healthy subjects and ten diabetic neuropathic subjects were analyzed (see Table 1). The healthy subjects did not have any metabolic, cardiovascular and neurological disease. Neuropathic diabetic subjects were recruited among the outpatients of the Antidiabetic Unit of the University Hospital of Padova, Italy. All volunteers were asked to sign an informed consent form.

**Table 1:** Descriptive information of control and neuropathic subjects

	**Sex** **n°**	**Age** **[years]**	**Normal Foot [%]**	**Claw Foot** **[n°]**	**Flat Foot [n°]**	**Heel Position** **[n°]**	**%BMI** **[kg/m^2^]**	**Walking Speed** **[m/s]**	**ND**
C	8 M 2 F	61.8 ± 4.3	3	7	0	5 Valgus L 1 Valgus R 2 Valgus RL	24.1 ± 3	1 ± 0.1	/
									
D	7 M 3 F	64 ± 6.8	/	9	1	6 Valgus L 1 Valgus R 3 Valgus RL 1 Varus R	24 ± 2.8	1 ± 0.2	10 PN 1 AN 5 R 2 V 2 M

Control (C) and diabetic (D) population: sex, age, foot size, foot type (normal foot, claw foot, flat foot), heel position, bmi, mean walking speed (mean and SD). Normal foot = non flat and non cavus foot; R = right, L = left. RL= right and left, M = male, F = female. ND = neuropathy diagnosis: peripheral neuropathy (PN), autonomic neuropathy (AN), retinopathy (R), Vasculopathy (V), Microalbuminuria (M)

### Neuropathy diagnosis

Diagnosis of neuropathy was assessed through anamnesis and clinical evaluation [2,27]. The following global and sectorial morphological examinations were performed: foot typology (normal-flat-claw foot), valgus big toe, stiff big toe, clawed toes, V^th ^normal-adducted toe, plantar examination (callosity, soft corn, ulcers, heel ragas), dominant hand, dominant foot, dominant eye/complementary eye, varum-valgus heel (R and L) [23,24].

### Anatomical landmarks definition and marker placement

**Figure 1:**
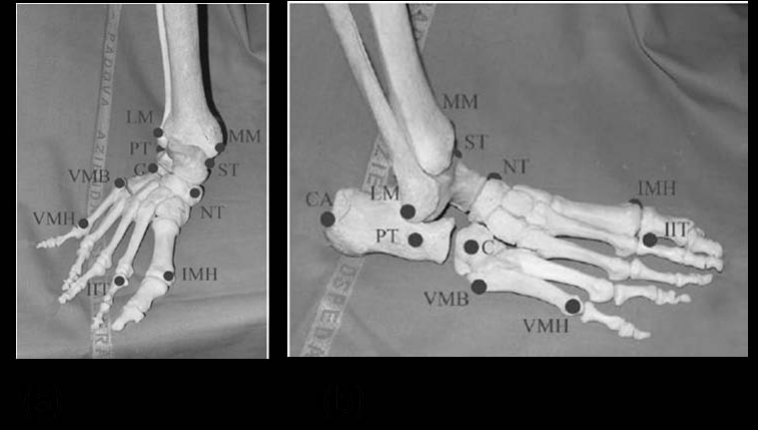
**The model anatomical landmarks identified on a skeleton foot (black circle), frontal (a) and lateral view (b)**.

Skin markers were attached through double sided tape on the ALs described in Table 2 and shown in Figure 1[24].

**Table 2:** Anatomical landmark description

**Anatomical Landmark**	**Segment**	**Description**
HF= Head of the Fibula	Tibia= tibia+ fibula	Proximal tip of the head of the fibula.
TT = Tibial Tuberosity		The most anterior border of the proximal extremity of tibial tuberosity.
LM = Lateral Malleolus		The lateral apex of the external malleolus.
MM = Medial Malleolus		Medial apex of the internal malleolus
ca= Calcaneus	Hindfoot= calcaneus and astragalus	Lower ridge of the calcaneus posterior surface.
PT = Peroneal Tubercle		Sitting with unloaded foot placed at 90° with respect to the sagittal axis of the fibula. Following the prolongation of inferior apex of the lateral malleolus, aligned with the longitudinal axis of the tibia, place the marker on the first bone prominence below the lateral mallleolus.
ST = Sustentaculum Talii		Sitting with unloaded foot placed at 90° with respect to the sagittal axis of the fibula. Following the prolongation of inferior apex of the medial malleolus, aligned with the longitudinal axis of the tibia, place the marker 2 cm under the distal border of the lateral malleolus: in correspondence of the last medial bone prominence before the medial muscle-tendon insertion of the calcaneus.
NT = Navicular Tuberosity	Midfoot= scaphoid, cuboid, 1^st^, 2^nd^, 3^rd ^cuneiform, 1^st^, 2^nd^, 3^rd ^metatarsus	Sitting with his unloaded foot placed at 90° with respect to the sagittal axis of the fibula. Ask the subject to relax the foot and find the proximal epyphisis of the 1^st ^metatarsal. Following the line between the proximal epyphisis of the 1^st ^metatarsal and the lower ridge of the calcaneus the first prominence that you palpate is the cuneiform and the second is the navicular. Once found the navicular bone on that line place the marker on the navicular following the line orthogonal to the floor on the interior side of the extensor longus of the allux (ask the subject to rise the allux to find the extensor longus).
C= Cuboid		Sitting with his unloaded foot placed at 90° with respect to the sagittal axis of the fibula. In correspondence of the proximal aspect of the 5^th ^metatarsal base following the direction of the tibia axis (orthogonal to the floor) place the marker on the first bone prominence you palpate on the cuboid.
VMB = Fifth Metatarsal Base		The most external surface of the base of the fifth metatarsus.
IMH = First Metatarsal Heads	Forefoot= 1^st^, 2^nd^,3^rd^,4^th^,5^th ^metatarsal heads and phalanxes (1^st^, 2^nd^,3^rd^,4^th^,5^th ^toes)	Head of the 1st metatarsus
VMH = Fifth Metatarsal Heads		Head of the Vth metatarsus
IIT = Proximal epiphysis of second toe phalanx		Choose the 2^nd ^ray with the left hand, and with the right hand move the proximal phalanx of the second toe in dorsi and plantarflexion; place 1 cm distal from the joint interstice.

### Bone embedded frames

The foot and ankle complex was divided into sub-segments. Relevant anatomical BEFs were defined for each segment and sub-segment as described in Table 3 following international conventions [28].

**Table 3:** Anatomical bone embedded frames

**SEGMENT**	**AXIS**	**JOINT COORDINATE SYSTEM**
Tibia	y	The two malleoli and the head of fibula define a quasi frontal plane, the y axis is parallel to the line connecting the midpoint between LM and MM and the projection of the tibial tuberosity (TT) on this plane with its positive direction upward.
	x	The line connecting lateral and medial malleoli (LM e MM) and y axis define a plane: x is orthogonal to that plane with its positive direction forward (obtained as product between the two above described lines).
	z	Product between axis x and y.
	Origin	Midpoint between LM and MM.
Hindfoot	z	Parallel to the line connecting ST and peroneal tubercle PT with its positive direction from left to right.
	y	The line connecting calcaneus (CA) and substentaculum talii (ST) and the z axis define a plane: y axis is orthogonal to that plane with its positive direction upward (obtained as product between the two above described lines).
	x	Product between axis y and z.
	Origin	CA.
Midfoot	z	Parallel to the line connecting NT and C with its positive direction from left to right.
	y	The line connecting (NT), and fifth metatarsal base (VMB) and z axis define a plane: y axis is orthogonal to that plane with its positive direction from proximal to distal segment (obtained as product between the two above described lines).
	x	Product between axis y and z.
	Origin	Midpoint between NT and C.
Forefoot	z	Parallel to the line connecting IMH and VMH with its positive direction from left to right.
	y	The line connecting VMH and IIT and the z axis define a plane: y is orthogonal to the plane with its positive direction upward (obtained as product between the two above described lines).
	x	Product between y and z.
	Origin	Midpoint between IMH e VMH.
Foot	z	Parallel to the line connecting IMH e VMH with its positive direction from left to right.
	y	CA, IMH and VMH define a plane; the line connecting IIT and CA belong to a plane perpendicular to the previous one; z axis is parallel to the line intersection between the two planes with its positive direction forward.
	x	Product between axis y and z.
	Origin	CA.

### Elementary movements

The ability of the model to distinguish adjacent segments relative movements was tested on a subject's foot by performing the set of passive elementary movements described in Table 4 [see Additional file 1] and by reconstructing the relevant kinematics. In order to reduce intra-operator variability the experiment was repeated three times by the same operator.

**Table 4:** Control and neuropathic group foot sub-segments' and ankle's joint rotations

	**Joint/Segments**	**Hindfoot-Tibia**			**Midfoot-Hinfoot**			**Forefoot-Midfoot**			**Ankle**		
													
	**Rotation [deg]**	**I/E**	**Int/Ext**	**D/P**	**I/E**	**Int/Ext**	**D/P**	**I/E**	**Int/Ext**	**D/P**	**I/E**	**Int/Ext**	**D/P**
C	Range	5.5	2.2	15.9	4.2	3.6	4.6	4.2	3.9	16.0	11.5	6.1	25.9
	SD min	2.3	1.0	1.8	1.5	1.1	2.0	1.3	0.8	3.7	1.6	1.1	1.3
	SD max	4.4	2.6	4.7	3.4	3.3	6.8	3.2	3.4	9.1	7.1	4.2	4.7
	SD mean	3.0	1.8	3.1	2.3	1.8	3.8	2.1	2.0	5.7	3.4	2.3	3.0
N	Range	11.3	12.7	3.4	9.1	1.8	9.3	16.2	9.9	55.2	16.2	12.5	48.8
	SD min	0.0	0.0	0.0	0.0	0.0	0.0	0.0	0.0	0.0	0.0	0.0	0.1
	SD max	1.9	4.6	2.9	3.8	7.2	3.6	9.9	6.4	16.7	3.6	9.9	25.1
	SD mean	0.5	0.8	1.04	0.5	0.7	0.8	0.6	0.7	1.6	0.7	0.6	1.7

C= control group; N= neuropathic group; Standard deviation = SD; Inversion-Eversion = I/E, Dorsi/Plantarflexion = D/P, Int/Ext Rotation= Internal/External

### Motor tasks

Each subject was assessed during both static and dynamic trials. During the static trial subjects were asked to assume an upright posture with their feet placed with ankles together, toes pointed 30 degrees apart and the arms along the body [23]. To ensure similar angles throughout the ensemble, a guides made of heavy cardboard have been placed between the performer's feet to set them at the correct angle. The performer lined his feet up along both arms of the footguide. In the dynamic trials subjects were asked to walk on the level at their normal speed of progression looking at a target placed at their eyes height. Three walking trials with full contact on each force plate were collected in order to determine each trial right and left gait cycle.

### Repeatability analysis

In order to verify the repeatability of the model three different tests were performed on both the pathological and normal subjects [29-32].

Test 1: all the markers were placed on the same subject during the same day by the same clinician (inter-trial variability).

Test 2: all the markers were placed on the same subject during two different sessions separated by several weeks (inter-day variability) by the same clinician.

Test 3: all the markers were placed on the same subject during the same session by five different clinicians appropriately trained in the same way by the same clinician (inter-session variability).

For each test, three walking trials per subject were acquired together with a static acquisition.

### Joint kinematics

The following model segments and joints relative motion were considered: motion of the ankle joint as complete foot vs. tibia, motion of the hindfoot vs. tibia, motion of the midfoot vs. hindfoot, motion of the forefoot vs. midfoot. Dorsi-plantarflexion (D/P) motion was considered as the distal segment rotation around the mediolateral axis of the proximal one, inversion-eversion (I/E) angle as the distal segment rotation around its anteroposterior axis, internal-external (Int/Ext) rotation as the segment rotation around the axis obtained as cross product between the other two axis [25]. Model segments and joints rotation angles were calculated as described in Table 3 according to Cardan convention.

### Neutral position

The static acquisitions were used to determine the analyzed joints neutral orientations.

### Skin artefacts

The static acquisition together with a specific algorithm was used to define each segment anatomical BEF, to minimize skin artifacts and to prevent errors related to markers occlusion. The algorithm, based on the hypothesis that every segment behaves like a rigid body, checks for the mutual distances between markers placed on the same anatomical segment during the walking trials comparing them to the values obtained through the static analysis. So far the distances between each marker belonging to the same segment were computed. In case of significant deviations the operator could decide either to correct the marker position through an interpolation procedure or, in the worst case, to exclude the trial from the analysis.

### Statistical analysis

One way ANOVA (Matlab software *anova1*) analysis of variance was used in order to compare diabetic kinematics to control group. One way ANOVA was performed on joint rotation angles relatively to specific gait cycle phases according to Perry definition [33]: 0-2% of gait cycle corresponds to initial contact, 0-10% to loading response, 10-30% to midstance, 30-50% to terminal stance, 50-60% to preswing, 60-73% to initial swing, 73-87% to midswing, 87-100% to terminal swing.

## Results

### Normative bands

**Figure 2:**
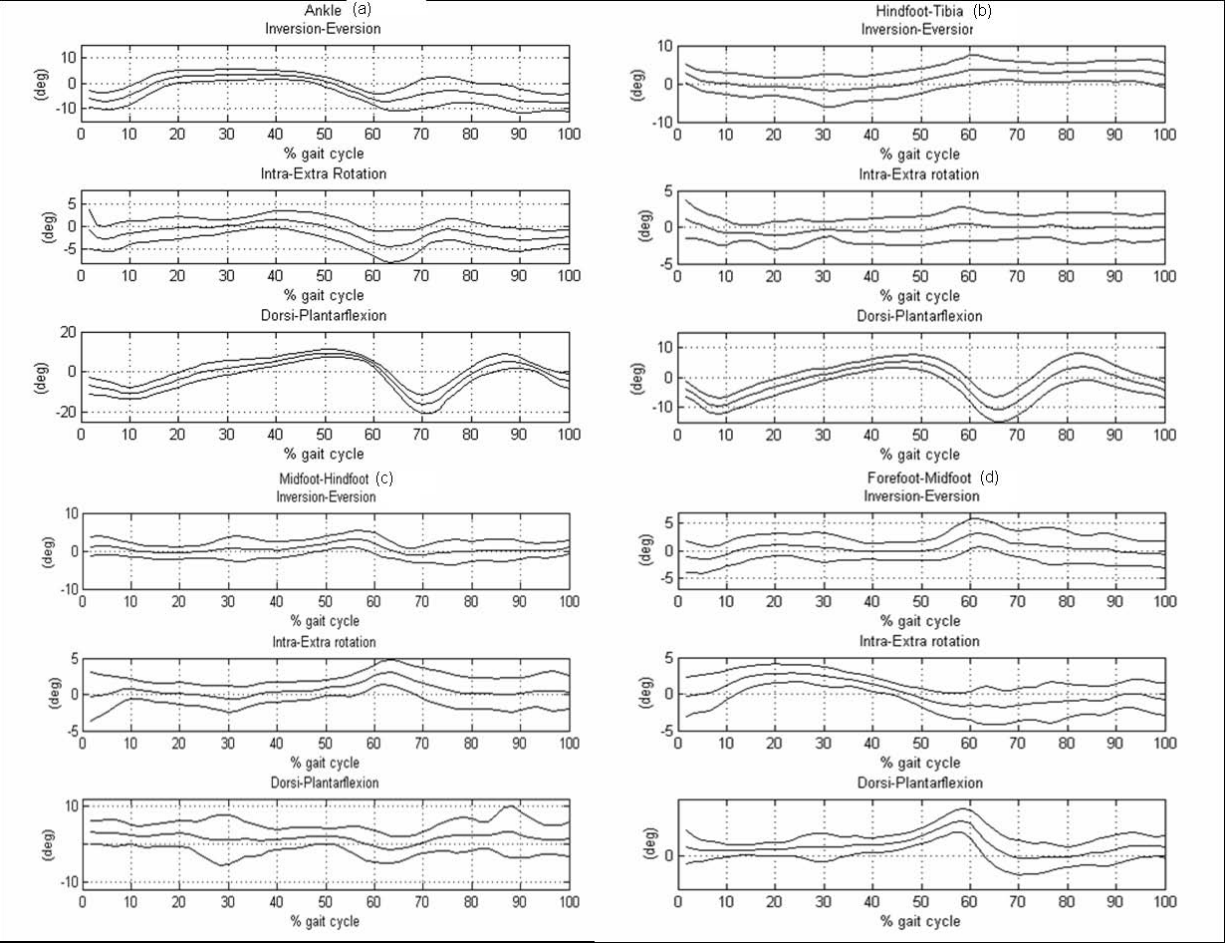
**Joint rotation normative bands (mean and 1 standard deviation (SD)) created using the data of ten healthy subjects**. (a) Ankle joint rotation, (b) hindfoot vs tibia rotation, (c), midfoot vs hindfoot rotation, (d) forefoot vs midfoot rotation.

Joint rotation normative bands (mean plus/minus one standard deviation (SD)) were generated (see Figure 2) using the data of the control group. Three representative strides per subject were selected.

Range of the control group joint and model segments rotation, together with maximum and minimum SD value were reported in Table 4.

### Repeatability analysis

The repeatability analysis results reported in Table 5 and [see Additional file 2] are expressed as mean, range and SD, Noonan coefficient of absolute variability values (Vabs) [29]. These have been compared with literature values and found comparable as reported respectively in Table 5[19,21,22] and in [see Additional file 2] [20,21,30,31]. The repeatability analysis carried out on pathological subjects yielded results comparable to those found in the control group in most of the joint and model segments rotation angles in terms of SD and Vabs.

**Table 5:** Variability analysis comparison with the literature

	**Inter-day variation [deg]**						**Inter-trial variation [deg]**			**Inter-session variation [deg]**	
	**Current study**		**Simon et Al.** [22]		**Carson et. Al.** [19]	**Stebbins et Al.** [21]	**Current study**	**Simon et Al.** [22]	**Carson et. Al.** [19]	**Current study**	**Simon et Al.** [22]
	Range	sd	Range	sd	sd	Sd	sd	sd	sd	sd	sd
Tibio-talar flexion	17.26	1.66	22.2	1.34	1.4	-	0.97	0.93	0.66	3.91	1.89
Subtalar inversion	13.46	2.61	10	3.38	3	-	1.22	0.8	0.68	2.03	3.2
Forefoot/midfoot supination	8.32	0.95	-	1.38	-	1.6	0.61	0.53	-	2.92	7.29
Forefoot/hindfoot abduction	14.24	1.57	9	2.54	4.3	2.4	0.59	0.55	0.57	2.23	3
Forefoot/midfoot dorsiflexion	24.27	2.89	-	-	-	2.7	0.78	-	-	3.2	-
Forefoot/ankle supination	12.06	1.59	11.5	1.35	3.3	3.1	0.63	0.74	0.59	2.36	3.3
Forefoot/ankle abduction	16.14	1.26	12	1.22	-	3.7	0.65	0.67	-	2.65	3,29

Variability analysis includes: inter-day variation, inter-trial variation, inter-tester variation. Mean and SD are obtained from a single subject among five examiners over the mean of three repetitions (inter-session variability), from a single subject and the same clinician during the same day over the mean of three repetitions (inter-trial variability), from a single subject and the same clinician during two different sessions separated by several weeks over the mean of three repetitions (inter-day variability). In [22] one subject was examined by five different testers and repeatedly by the same tester for five sessions. In [19] sixteen test sessions were completed with two testers assessing each of two healthy subjects independently over four days separated by a minimum of one week. In [21] fifteen healthy children were tested on three separate occasions. Visits were spaced between 2 weeks and 6 months apart, three representative strides from three separate trials were used for analysis from each session.

### Elementary movements

The mean values of the passive elementary movements were calculated and are reported in [see Additional file 1] and Table 6. The results of the elementary movements executed were used both to test the ability of the model to resolve clinically significant relative movements of the adjacent segments as well as to test the model precision. The results relative to the adjacent sub-segments motions displayed in Table 6 are expressed in mean, range and SD.

**Table 6:** Elementary movements' sub-segments and joint angles results

**Elementary Movements**	**Hindfoot - Tibia**			**Midfoot - Hindfoot**			**Forefoot - Midfoot**		
**[deg]**	**Mean**	**Range**	**SD**	**Mean**	**Range**	**SD**	**Mean**	**Range**	**SD**
Flexion	9.3	9.8	1.1	7.0	7.8	1.2	6.9	8.2	1.4
Extension	2.5	13.4	0.3	1.4	10.2	0.6	2.9	10.4	1.2
External Rotation	15.8	5.2	2.1	5.2	6.2	1.9	5.7	3.1	0.2
Internal Rotation	5.2	20.3	2.1	11.8	13.4	1.9	5.5	9.7	1.5
Inversion	8.1	1.3	0.1	10.1	12.2	3.8	2.1	3.6	1.3
Eversion	7.3	3.1	1.8	8.2	11.9	2.6	8.2	3.1	1.1

### Statistical analysis

Range, maximum and minimum SD value of the neuropathic group corresponding to each segment relative motion and joint rotation were reported in Table 4.

**Figure 3:**
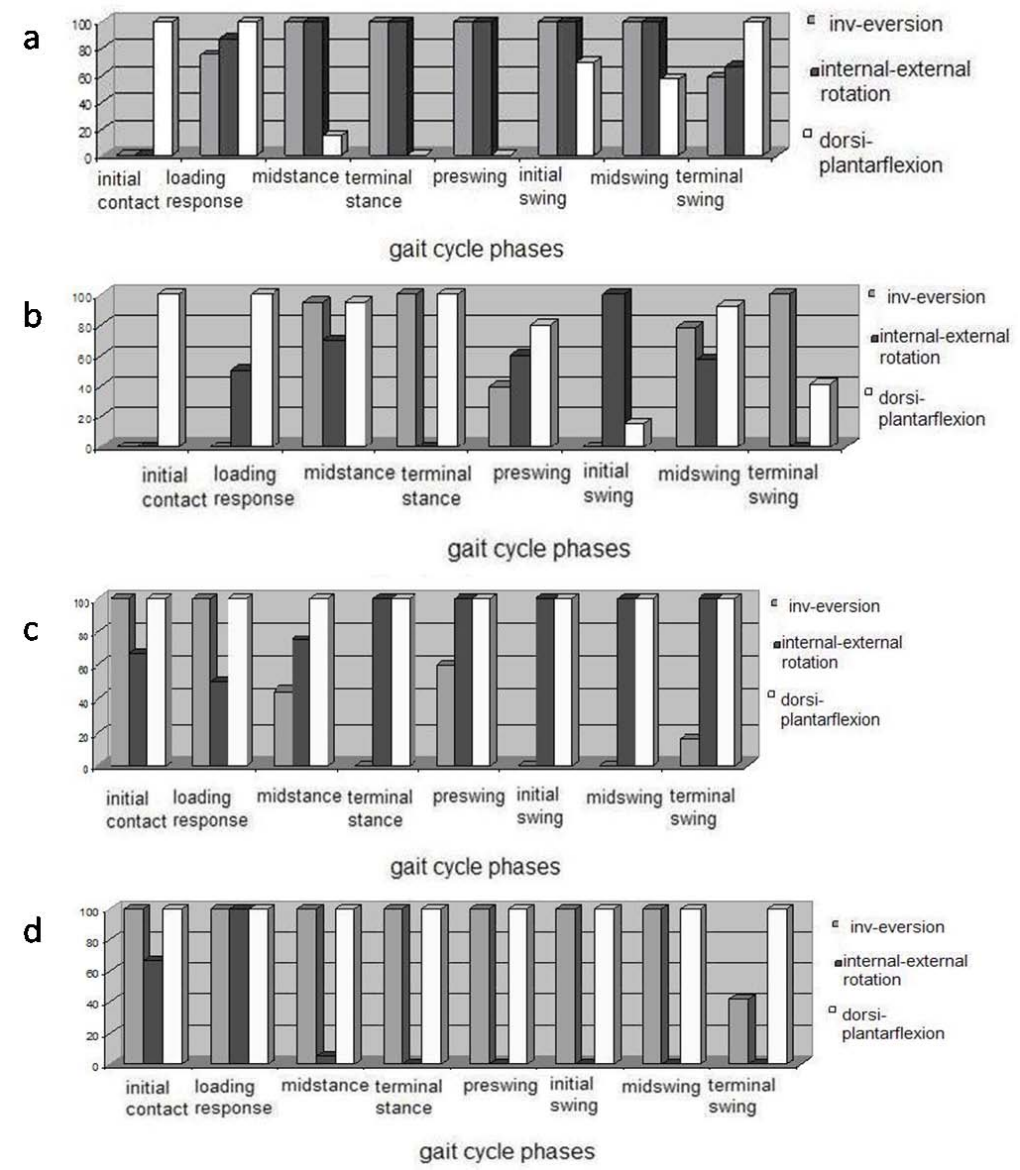
**Results of 1 way ANOVA analysis between neuropathic and control groups kinematics variables**. Each phase of the gait cycle percentage of frames with p < 0.05 have been reported for each joint rotation. (a) Ankle joint rotation, foot subsegments rotation: (b) hindfoot vs tibia rotation, (c) midfoot vs hindfoot rotation, (d) forefoot vs midfoot rotation.

One way ANOVA was performed on joint rotation angles (Figure 3) in order to identify significant variables in neuropathic populations. Results were expressed as percentage of frame (relatively to specific gait cycle phases) where statistically significant differences (p < 0.05) were found between joint rotations describing the amount of biomechanical impairment observed relatively to the specific movement that each joint was performing during the gait.

## Discussion

Even though various biomechanical models have been developed so far to study the properties and behaviour of the foot [14,18,19,21,22], the present study focuses on developing a methodology for the functional assessment of the foot-ankle complex and for the definition of a functional model of the diabetic neuropathic foot.

A method for capturing forefoot, midfoot and hindfoot motion during different gait tasks have been proposed. The model includes tibia and fibula, hindfoot, midfoot and forefoot, and allows investigation of 3-dimensional foot and ankle kinematics through stereophotogrammetry. A new model has been generated since available foot protocols were not suitable for this type of analysis [18,19,21,22,26]. One important limitation of the literature was that the 3 planar motion of the midfoot was not evaluated. As the diabetic foot disease accounts for midfoot structural polymorphism which commonly leads to plantar ulceration [34,35], the authors believe that a suitable model to describe the diabetic foot biomechanics should perform 3D midfoot kinematic analysis. Furthermore, this was confirmed by the results reported in Figure 3 where, the diabetic group has statistically significant differences in midfoot kinematic parameters over a large part of the gait cycle. Nevertheless the forefoot should be considered entirely and not represented by a single toe as the hallux [36] because it is considered the high risk zone for plantar ulcer formation [4,36]. This was confirmed by the results reported in Figure 3 where, the diabetic group showed statistically significant differences in forefoot kinematic parameters over the full gait cycle in the sagittal and coronal planes, and in the 50% of gait cycle in the transversal plane. Furthermore in the literature has been reported that in diabetic patients, changes in weight bearing patterns are linked to limited joint mobility that occurs mostly at metatarsophalangeals and subtalar joints. Nevertheless the location of forefoot plantar ulcers in diabetic subjects has been demonstrated to be highly correlated with rearfoot alignment [4]. In addition to the different types of mechanisms of excessive pressure loading, abnormal alignment of the foot also affects pressure loading on the foot. Finally patients with an uncompensated forefoot varus or forefoot valgus (inverted or everted forefoot) had ulcers located at the first or fifth metatarsal head. Similarly, an inverted heel position has been associated with lateral ulcers, whereas an everted heel position has been associated with medial ulcers [36]. So far the authors believe that a technique for the measurement of rearfoot-forefoot-midfoot structures alignment is needed in understanding the aetiology of diabetic foot ulcers. Finally, the triplanar orientation of the joint axis allows for movement in all three body planes and thus provides a mechanism for compensatory motion if there is presence of structural anomalies in the foot [4], which is indeed the case of the diabetic foot.

Protocols which adopt rigid array of markers and procedures which implies calibration techniques [13-15] where not adopted because they present as major disadvantage the time required for each anatomical landmark calibration trial. Furthermore when errors due to skin artefacts affect protocols using mounting plates, it is difficult to identify the relative contribution of each individual marker, as the errors affect the complete cluster as a whole. Therefore we choose direct skin marker placement on ALs even though these are more subject to errors due to skin artefacts and markers misplacement [26]. An extensive review of the problem is given in Leardini et al. 2005 [37]. This protocol tries to prevent these errors by using the above described algorithm with a static calibration and by controlling for markers occlusion.

The ALs were selected in order to be easily palpated and identified. The location of the ALs was chosen so that BEFs were directly defined with no need of technical frames definition [15].

As suggested by Baker [26] a foot model have been applied to a pathologic population, even though in this specific case one of the existing models could not be adopted as was previously done by Woodburn [38] for the reasons reported above. In the present work, ten neuropathic subjects have been evaluated and results of this analysis showed major statistically significant differences between the two populations both in the forefoot vs midfoot and midfoot vs hindfoot dorsi-plantarflexion (100% of frame of the gait cycle). Also important statistically significant differences were observed in midfoot vs hindfoot internal-external rotation (90% of frame of the gait cycle), in forefoot vs midfoot inversion-eversion, in ankle internal-external rotation (96% of frame of the gait cycle) and inversion-eversion (92% of frame of the gait cycle). Thus to confirm the validity of a similar approach in order to assess diabetic neuropathics' biomechanics impairment.

An important step in assessing the effectiveness of gait analysis is to establish the precision of the data collection [32] and the accuracy in determining the anatomical landmarks and joint embedded frames definition. An effort in this direction is documented in Table 2 and 3 were a detailed description of ALs, and BEFs together with instruction for marker placement can be found. It is, of course, important to evaluate the major sources of variation in gait analysis (true variation in the subject's gait and artefact from the measurement procedure) [32]. We therefore need an estimate of the expected variability in joint rotation angles estimation. This is important when, for example, comparing a patient data against normative standards - we need to know how much difference is significant. Normal biological variation affects kinematics data since subjects never walk in the exact same way in every trial, therefore variability introduced by the subject within a test session were examined. Variability is also introduced by the measurement procedure by means of anatomical landmarks identification and skin movement artefact. Therefore joint rotation angles variability due to differences between clinicians, and by the subject within and between test days were examined [30-32]. Three walking trials per subject were acquired together with a static acquisition. The same procedure was applied both to normal and pathological subject, in order to check the feasibility of this approach onto diabetic subjects.

Repeatability has been assessed by the mean, range and SD values of model segments and joint rotation angles, together with Vabs coefficient of Noonan [29], following the methodology proposed by Schwartz [32]. The results showed the suitability of this method as they were found comparable with similar studies [19,21,29,21,22,31] and this gives strength to the present work. The repeatability analysis on the pathological subject shows results comparable to the normal one in terms of SD and Vabs in most of the rotation angles, which asses the suitability of this protocol to this type of patients.

Based on the results reported in Table 5 and [see Additional file 2], we can assess that the model has been tested for repeatability therefore anatomical landmark identification can be considered feasible.

The elementary movements were used in order to check the ability of the model to measure sub-segments rotations. The range, mean and SD values of the angles obtained by executing passive movements of the foot allowed us to test the suitability of the chosen reference systems and angles definition. We think this is in fact the only possible way to quantitatively assess the capability of the model of measuring correctly model segments rotations. Since the possibility of executing elementary movements of each model segment is still under study in our laboratory, model segments rotations were obtained by performing full foot elementary passive movements. Then the movement of each segment component was obtained by the model. The rotations relative to model segments are considered clinically acceptable [39].

## Conclusion

A method for assessing foot subsegment three-dimensional kinematics have been obtained leading to results clinically consistent [39] and repeatable. The model has been tested for repeatability and shows results which agree with previous literature findings on kinematics data variability [19,25,29,31,32].

Even though this study was applied to a limited number of patients, the proposed protocol appears to be clinically promising since it shows a good compliance by the patients. Indeed the neuropathic subject repeatability analysis shows results comparable with normal subjects. The marker set has been included in a full body protocol and has been implemented in routine clinical gait analysis of diabetic patients together with the simultaneous analysis through plantar pressure platforms and force plates [24]. The results are considered sufficiently repeatable to guarantee the clinical application of the protocol.

## Competing interests

Each of the authors has read and concurs with the content in the final manuscript.

The contributing authors guarantee that this manuscript has not been submitted, nor published elsewhere.

Each of the authors declare that don't have any financial and non-financial competing interests

## Authors' contributions

Each of the authors has read and concurs with the content in the final manuscript.

ZS, GC, GG, AA, SC and CC participated in conceiving the study. ZS, GC, GG, AA and CC participated in its design and coordination and carried out the drafting of the manuscript. SC and GD helped to draft the manuscript. ZS carried out the experimental part of the study relatives to the motion analysis data collection and carried out and coordinated the data analysis. GD, AF and PD participated to the experimental part of the study relatives to the motion analysis data collection and performed some of the data analysis. GC and GG carried out the experimental part of the study relatives to the Neuropathy Diagnosis and participated to the motion analysis data collection.

## Supplementary Material

Additional file 1 **Elementary movements description**. Detailed description of the elementary movements. Click here for file

Additional file 2 **Variability analysis on normal and pathological subjects for inter-session, inter-trial and inter-day variation**. Variability analysis on normal and pathological subjects for inter-session, inter-trial and inter-day variation. Results are expressed as mean, sd and 'mean absolute variability' (Noonan coefficient of absolute variability = Vabs [29]) of the kinematics variables. Vabs coefficients are obtained from a single subject among five examiners over the mean of three repetitions (inter-session variability), from a single subject and the same clinician during the same day over the mean of three repetitions (inter-trial variability), from a single subject and the same clinician during two different sessions separated by several weeks over the mean of three repetitions (inter-day variability). Values are all in degrees. Results are compared with literature values ([20,29-31]). In [30] and [31] coefficients were obtained from a single subject among 24 examiners and 12 sites, respectively before a training program, [30], and after; from 11 subjects among 4 sites [29], and from a single subject among five examiners over the mean of three repetitions [29]. Click here for file

## References

[B1] Fedele D, Comi G, Costelli C, Cucinotta D, Feldman EL, Ghirlanda G, Greene DA, Negrin P, Santeusanio F (1997). Italian Diabetic Neuropath Committee, A multicenter study on the prevalence of diabetic neuropathy in Italy. Diabetes Care.

[B2] Lavery AL, Armstrong DG, Vela AS, Quebedeaux TL, Fleischli JG (1998). Pratical criteria for screening patients at high risk for diabetic foot ulceration. Arch Intern Med.

[B3] Andersen H, Gadeberg PC, Brock B, Jakobsen J (1997). Muscolar atrophy in diabetic neuropathysterelogical magnetic resonance imaging study. Diabetologia.

[B4] Bevans J (1992). Biomechanics and plantar ulcers in diabetes. Foot.

[B5] Abbott CA, Vileikyte L, Williamson S, Carrington AL, Boulton AJ (1998). Multicenter study of the incidence of and predictive risk factor for diabetic neuropathic foot ulceration. Diabetes Care.

[B6] Giacomozzi C, Caselli A, Macellari V, Giurato L, Lardieri L, Uccioli L (2002). Walking strategy in diabetic patients with peripheral neuropathy. Diabetes Care.

[B7] Andersen H, Jakobsen J (1999). Motor function in diabetes. Diabetes Rev.

[B8] Uccioli L, Giacomini PG, Monticone G, Magrini A, Durola L, Bruno E, Parisi L, Di Girolamo S, Menzinger G (1995). Body sway in diabetic neuropathy. Diabetes Care.

[B9] D'Ambrogi E, Giurato L, D'Agostino MA, Giacomozzi C, Macellari V, Caselli A, Uccioli L (2003). Contribution of plantar fascia to the increased forefoot pressures in diabetic patients. Diabetes Care.

[B10] Cavanagh PR, Simoneau GG, Ulbrecht JS (1993). Ulceration, unsteadiness, and uncertainty: the biomechanical consequences of diabetes mellitus. Journal of Biomechanics.

[B11] Shaw JE, Van Schie CHM, Carrington AL, Abbott CA, Boulton AJM (1998). An analysis of dynamic forces transmitted through the foot in diabetic neuropathy. Diabetes Care.

[B12] Mueller MJ, Sinacore DR, Hoogstrate S, Daly L (1994). Hip and Ankle walking Strategies: Effect on Peack Plantar Pressures and Implications for Neuropathic Ulceration. Arch Phys Med Rehab.

[B13] Leardini A, Benedetti MG, Catani F, Simoncini L, Giannini S (1999). An anatomically based protocol for the description of foot segment kinematics during gait. Clinical Biomechanics.

[B14] Jenkyn TR (2002). Motion of the ankle complex and forefoot twist during walking and medial direction changes [abstract]. Gait and Posture.

[B15] Cappozzo A, Catani F, Della Croce U, Leardini A (1995). Position and orientation in space of bones during movement: anatomical frame definition and determination. Clinical Biomechanics.

[B16] Davis RB, Ounpuu S, Tyburski D, Gage JR (1991). A gait data collection and reduction technique. Human Movement Sciences.

[B17] Benedetti MG, Catani F, Leardini A, Pignotti E, Giannini S (1998). Data management in gait analysis for clinical applications. Clinical Biomechanics.

[B18] Simon J (2000). A multi-segmented foot model. Gait and Posture.

[B19] Carson MC, Harrington ME, Thompson N, O'Connor JJ, Theologis TN (2001). Kinematic analysis of a multi-segment foot model for research and clinical applications a repeatability analysis. Journal of Biomechanics.

[B20] Leardini A, Sawacha Z, Paolini G, Ingrosso S, Nativo R, Benedetti MG (2007). A new anatomically based protocol for gait analysis in children. Gait and Posture.

[B21] Stebbins J, Harrington M, Thompson N, Zavatsky A, Theologis T (2006). Repeatability of a model for measuring multi-segment foot kinematics in children. Gait and Posture.

[B22] Simon J, Doederlein L, McIntsh AS, Metaxiotis D, Bock HG, Wolf SI (2006). The Heidelberg foot measurement method: Development, description and assessment. Gait and Posture.

[B23] Bourdiol JR (1980). Pied et statique.

[B24] Sawacha Z, Cristoferi G, Guarneri G, Corazza S, Donà G, Facchinetti A, Denard C, Sommavilla M, Zaccaria M, Avogaro A, Cobelli C, Belmonti V, Cluccini M, Sgandurra G (2005). A method for the simultaneous assessment of gait and posture. Proceedings of Italian Society of Movement Analysis in Clinic: 26-29 October Pisa, Italy.

[B25] Sawacha Z, Cristoferi G, Mattioli N, Guarneri G, Corazza S, Avogaro A, Cobelli C A 3d four segment anatomy based protocol for the description of foot and ankle kinematics. Proceedings of Gait and Clinical Movement Analysis society: 6-9 April 2005; Portland.

[B26] Baker R, Robb J (2006). Foot models for clinical gait analysis. Gait and Posture.

[B27] Perkins BA, Bril V (2003). Diabetic neuropathy: a review emphasizing diagnostic methods. Clin Neurophysiol.

[B28] Wu G, Siegler S, Allard P, Kirtley C, Leardini A, Rosenbaum D, Whittle M, D'Lima DD, Cristofolini L, Witte H, Schmid O, Stokes I (2002). ISB recommendation on definitions of joint coordinate system of various joints for the reporting of human joint motion-part I: ankle, hip and spine. Journal of Biomechanics.

[B29] Noonan KJ, Halliday S, Browne R, O'Brien S, Kayes K, Feinberg J (2003). Interobserver variability of gait analysis in patients with cerebral palsy. J Pediatr Orthop.

[B30] Gorton G, Hebert D, Goode B (2001). Assessment of the kinematic variability between 12 Shriners Motion Analysis Laboratories. Gait and Posture.

[B31] Gorton G, Hebert D, Goode B (2002). Assessment of the kinematic variability between 12 Shriners Motion Analysis Laboratories. Part 2. Short term follow up. Gait and Posture.

[B32] Schwartz M, Trost J, Wervey R (2004). Measurement and management of errors in quantitative gait data. Gait and Posture.

[B33] Perry J (1992). Gait Analysis, Normal and Pathological Function.

[B34] Sinacore DR, Bohnert KL, Hastings MK, Johnson JE (2008). Mid foot kinetics characterize structural polymorphism in diabetic foot disease. Clinical Biomechanics.

[B35] Van Schie CHM (2005). A Review of the biomechanics of the diabetic foot. Lower Extremity Wounds.

[B36] MacWilliams B, Cowley M, Nicholson D (2003). Foot kinematics and kinetics during adolescent gait. Gait and Posture.

[B37] Leardini A, Chiari L, Della Croce U, Cappozzo A (2005). Human movement analysis using stereophotogrammetry, Part 3: soft tissue artefact assessment and compensation. Gait and Posture.

[B38] Woodburn J, Nelson KM, Siegel KL, Kepple TM, Gerber LH (2004). Multisegment foot motion during gait: proof of concept in rheumatoid arthritis. J Rheumatol.

[B39] Kapandji IA, Kandel MJ, Churchill Livingstone (1988). Lower Limb. In the Physiology of the Joints.

