# Supplementary material for: Characterizing multisegment foot kinematics during gait in diabetic foot patients
Source: J Neuroeng Rehabil. 2009 Oct 23;6:37. doi: 10.1186/1743-0003-6-37 (PMC2775023; doi:10.1186/1743-0003-6-37)
Supplement: Additional file 1 — Elementary movements description. Detailed description of the elementary movements. [file 1743-0003-6-37-S1.PDF]

Elementary movements description

Elementary movements	Name	Description
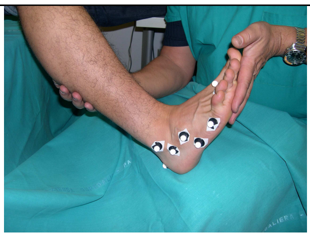	**Dorsiflexion**	**The subject is sitting with the unloaded leg at 45° to the floor. Place the right hand facing up posteriorely to the tibia on the shank. Place the left hand in contact with the plantar surface in correspondence to the metatarsal heads and push the foot upward toward the maximum flexion.**
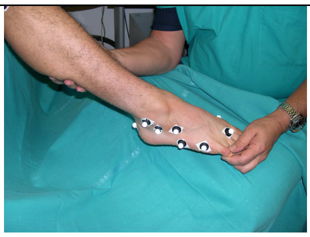	**Plantarflexion**	**The subject is sitting with the unloaded leg at 45° to the floor. Place the right hand facing up posterior to the tibia on the shank. Place the left hand in contact with the first phalanx of the toes from 1st to 5th. Pull the toes without changing the angle between metatarsal and phalanxes so that the complete foot will follow the applied motion.**
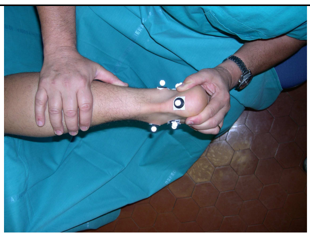	**Internal Rotation (valgus heel)**	**The subject leans down with the unloaded leg at 45° to the ceiling. Place the right hand facing down posteriorely to the tibia on the shank. Place the left hand in contact with the posterior aspect of calcaneus without touching or moving the hindfoot markers. Pull the calcaneus laterally with the left hand (valgus).**
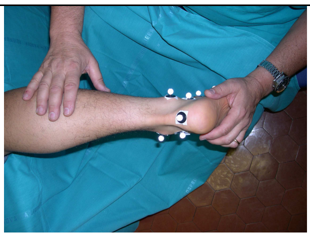	**External Rotation (varus heel)**	**The subject leans down with the unloaded leg at 45° to the ceiling. Place the right hand facing down posteriorely to the tibia on the shank. Place the left hand in contact with the posterior aspect of calcaneus without touching or moving the hindfoot markers. Pull the calcaneus medially with the right hand (varus).**
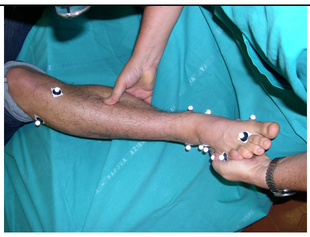	**Inversion**	**The subject is sitting with the unloaded leg at 45° to the floor. Place the right hand anteriorely on the tibia at 1/3 of the distance between lateral malleoli and head of fibula. Place the left hand, facing up, onto the plantar surface in order to block the motion of 1st and 5th metatarsal. Turn the foot with the left hand provoking an inversion movement.**
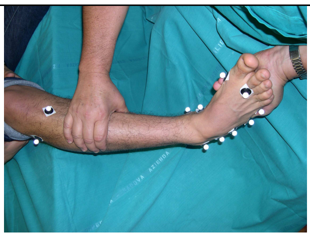	**Eversion**	**The subject is sitting with the unloaded leg at 45° to the floor. Place the right hand anteriorely on the tibia at 1/3 of the distance between lateral malleoli and head of fibula. Place the left hand, facing up, onto the plantar surface in order to block the motion of 1st and 5th metatarsal. Turn**

		**the foot with the left hand provoking an eversion movement.**
