# Supplementary material for: Characterizing multisegment foot kinematics during gait in diabetic foot patients
Source: J Neuroeng Rehabil. 2009 Oct 23;6:37. doi: 10.1186/1743-0003-6-37 (PMC2775023; doi:10.1186/1743-0003-6-37)
Supplement: Additional file 2 — Variability analysis on normal and pathological subjects for inter-session, inter-trial and inter-day variation. Variability analysis on normal and pathological subjects for inter-session, inter-trial and inter-day variation. Results are expressed as mean, sd and 'mean absolute variability' (Noonan coefficient of absolute variability = Vabs [29]) of the kinematics variables. Vabs coefficients are obtained from a single subject among five examiners over the mean of three repetitions (inter-session variability), from a single subject and the same clinician during the same day over the mean of three repetitions (inter-trial variability), from a single subject and the same clinician during two different sessions separated by several weeks over the mean of three repetitions (inter-day variability). Values are all in degrees. Results are compared with literature values ([20,29-31]). In [30] and [31] coefficients were obtained from a single subject among 24 examiners and 12 sites, respectively before a training program, [30], and after; from 11 subjects among 4 sites [29], and from a single subject among five examiners over the mean of three repetitions [29]. [file 1743-0003-6-37-S2.PDF]

Variability analysis on normal and pathological subjects for inter-session, inter-trial and inter-day variation

SEGMENTS		INTER-SESSION							INTER-TRIAL						INTER-DAY					
		Normal				Pathological			Normal			Pathological			Normal			Pathological		
		mean	sd	Vabs	Vabs Literature [20] [29] [30] [31]	mean	sd	Vabs	mean	sd	Vabs	mean	sd	Vabs	mean	sd	Vabs	mean	sd	Vabs
Hindfoot Tibia	I/E	1.0	2.0	0.2	23 [24]<br>5.8 [21]	-0.7	5.8	1.7	1.0	1.2	0.9	1.2	2.5	0.5	1.0	2.6	2.2	-4.0	-8.1	3.8
	Int/Ext	2.5	2.0	1.6		-1.9	4.5	1.4	2.5	1.0	1.4	-8.4	1.1	3.3	2.5	1.5	10.3	-4.7	-9.5	0.9
	D/P	-1.2	2.9	0.4	12.1 [31]<br>6.1 [32]<br>12 [24]<br>4.7 [21]	0.0	4.9	1.6	-1.2	1.0	0.4	-3.2	4.5	1.5	-1.2	1.7	6.4	1.4	2.8	1.8
Midfoot Hindfoot	I/E	2.5	1.9	0.6		1.2	1.2	0.5	2.5	1.1	0.8	-0.1	0.8	0.8	2.5	1.5	0.2	0.4	0.8	0.4
	Int/Ext	4.3	1.9	2.5		7.0	3.9	1.1	4.4	1.1	0.7	5.4	5.7	3.7	4.3	1.7	10.7	7.4	1.5	5.1
	D/P	1.2	2.4	2.7		-5.9	3.0	0.1	1.2	1.0	2	-7.4	1.6	2.3	1.2	1.9	15	-8.1	-1.6	1.8
Forefoot Midfoot	I/E	-1.7	2.9	0.1		-4.7	1.3	0.6	-1.6	0.6	0.4	-3.8	1.8	0.7	-1.7	1.0	1.7	-3.6	-7.3	1.1
	Int/Ext	4.9	1.6	0		-0.9	1.0	2.6	4.9	0.3	1.7	-6.3	2.4	2.1	4.9	1.1	0	-8.2	-1.6	4
	D/P	3.3	3.2	2.1		7.3	7.8	2.1	3.1	0.8	1.5	3.0	4.8	-1.9	3.4	2.9	1.7	2.3	4.6	5.1

Results are expressed as mean, sd and 'mean absolute variability' (Noonan coefficient of absolute variability = Vabs [29]) of the kinematics variables. Vabs coefficients are obtained from a single subject among five examiners over the mean of three repetitions (inter-session variability), from a single subject and the same clinician during the same day over the mean of three repetitions (inter-trial variability), from a single subject and the same clinician during two different sessions separated by several weeks over the mean of three repetitions (inter-day variability). Values are all in degrees. Results are compared with literature values ([20] [29] [30] [31]). In [30] and [31] coefficients were obtained from a single subject among 24 examiners and 12 sites, respectively before a training program, [30], and after; from 11 subjects among 4 sites [29], and from a single subject among five examiners over the mean of three repetitions [29].
